# Mechanisms of caries induced by sugars: a narratives review from microbial metabolism to oral ecological imbalance and public health strategies for caries prevention

**DOI:** 10.3389/fcimb.2026.1834886

**Published:** 2026-06-23

**Authors:** Anqi Zhang, Jielin Yang, Xiaozhong Wang, Botakoz Xehesbek, Jing Zhang, Xiao Hu, Bin Zhang, Ruizhe Huang

**Affiliations:** 1 Key Laboratory of Shaanxi Province for Craniofacial Precision Medicine Research, College of Stomatology, Xi’an Jiaotong University, Xi’an, China; 2 Clinical Research Center of Shaanxi Province for Dental and Maxillofacial Diseases, Center of Oral Public Health, College of Stomatology, Xi’an Jiaotong University, Xi’an, China; 3 Department of Pediatric Dentistry 1, Jinan Stomatological Hospital, Jinan, China; 4 Department of Stomatology, Xianyang Central Hospital, Xianyang, China

**Keywords:** caries prevention strategies, cariogenic mechanisms, dental caries, oral microbiome, sugars

## Abstract

Dental caries is defined as a chronic, multifactorial disease characterized by the demineralization of dental hard tissues resulting from the acid production by oral microbial communities metabolizing dietary sugars. The ingestion of sugars is a pivotal ecological factor in the progression of caries, with mechanisms that extend beyond merely providing substrates for cariogenic bacteria. This review explores the influence of sugars on the metabolism, adhesion, biofilm formation, and interspecies interactions of oral microorganisms, with a particular focus on species such as *Streptococcus mutans*, Lactobacilli, *Actinomyces*, and *Candida albicans*. The disruption of the oral microbiome balance by these sugars initiates and promotes the process of caries. The review comprehensively summarizes contemporary public health strategies for caries prevention based on microbial ecological theories, including the limitations of sugar intake, fluoride application, probiotics, and ecological management, assessing their effectiveness and challenges. The objective of this study is to establish a theoretical framework and practical guidelines for the precise prevention of dental caries.

## 1 Introduction

Dental caries, commonly referred to as tooth decay, is a highly prevalent chronic disease affecting populations worldwide (Pitts et al., 2021). Its etiology is closely associated with dietary habits, particularly the consumption of fermentable carbohydrates. Sugar broadly encompasses monosaccharides and disaccharides, with free sugars-such as sucrose, fructose, and glucose-being the most cariogenic (Moynihan, 2016). The World Health Organization (WHO) defines free sugars as monosaccharides and disaccharides that are added to foods and beverages or naturally present in honey, syrups, fruit juices, and fruit juice concentrates (WHO, 2002). The cariogenic potential of these sugars depends not only on their type but also on the frequency of intake and host-related factors.

Contemporary understanding recognizes dental caries as a biofilm-mediated and ecology-driven disease, rather than the result of a single pathogen. According to the ecological plaque hypothesis, frequent sugar exposure leads to sustained acid production and a reduction in plaque pH, selecting for acidogenic and aciduric microorganisms and driving a shift in the oral microbiome from symbiosis to dysbiosis (Lamont et al., 2018; Diaz-Garrido et al., 2020). This ecological imbalance, characterized by the enrichment of acid-tolerant species, underpins the initiation and progression of carious lesions.

The type, frequency, and quantity of sugar intake are directly related to caries development, with frequency being possibly more important than quantity (Van Loveren, 2019). Scholars have established through birth cohort studies and data analysis that there is a positive correlation between sugar intake and caries incidence, with higher frequencies and quantities of sugar consumption increasing the risk of caries and DMFT (Decayed, Missing, and Filled Teeth) values (Echeverria et al., 2022; Hong et al., 2018; Barrington et al., 2019; Olczak-Kowalczyk et al., 2016; Huang et al., 2023). The WHO recommends limiting daily sugar intake to less than 5% of total energy intake, approximately 25 grams, to mitigate the risk of sugar-related diseases (Huang et al., 2023). Sugars not only serve as substrates for cariogenic bacteria to produce acid but also alter microbial composition, shifting it towards a more cariogenic profile (Lamont et al., 2018). A study conducted in Pelotas demonstrated a positive correlation between sugar intake and the incidence of early childhood caries (ECC) (Echeverria et al., 2022). Higher sugar intake was associated with increased DMFT values in an investigation of sugar intake and caries prevalence among children in England (Hong et al., 2018). A study in Australia also revealed a positive correlation between sugar consumption and dental caries (Barrington et al., 2019). A comprehensive statistical analysis in Poland concluded that a 1 kg/year increase in sugar intake corresponded to a 1% rise in caries frequency and a 0. 2 increased in DMFT scores (Olczak-Kowalczyk et al., 2016). The physical and chemical forms of dietary sugars have changed significantly in recent decades, with important implications for caries risk.

The fundamental mechanism by which sucrose contributes to the development of dental caries involves the metabolic activity of cariogenic bacteria, such as *Streptococcus mutans*, which ferment sugars to produce extracellular polysaccharides (EPS) and organic acids. These acids reduce the pH level in the oral cavity, resulting in dysbiosis of the plaque microbiome and demineralization of tooth enamel. Glucose and fructose contribute significantly to acid production and microbial dysbiosis, while lactose, although less cariogenic, poses risks under certain dietary and oral conditions. Recent advancements in microbiome research have revealed a more complex interplay between sugar intake and oral microbial ecology, emphasizing the role of sugar not only as a substrate for acid production but also as a modulator of microbial community dynamics within the dental biofilm (Du et al., 2020). The consequences of this microbial imbalance are not limited to localized dental caries, existing evidence suggests a link between dental caries and cardiovascular diseases such as hypertension (Natarajan et al., 2025). Therefore, it is essential to understand the biochemical and ecological mechanisms by which sugar influences oral health in order to develop effective public health strategies aimed at preventing tooth decay.

In light of these considerations, this review provides a comprehensive overview of the biochemical pathways involved in sugar metabolism within the oral cavity. The focus was on the roles of key cariogenic bacteria and their interactions within the microbial community. It also assessed how these insights can inform public health initiatives aimed at reducing sugar intake and promoting oral health. And we also discussed multifaceted preventive strategies that leverage knowledge of microbial ecology, dietary habits and community health policies to combat the global burden of dental caries. This review adopts an integrated ecological and functional perspective to examine the role of dietary sugars in caries development. Specifically, it links sugar exposure patterns with microbial metabolic pathways, biofilm ecology, and translational prevention strategies within a unified framework. By emphasizing functional traits, ecological interactions, and emerging research directions, this review aims to provide a more comprehensive and forward-looking understanding of cariogenesis.

While previous systematic reviews have established the dose-response relationship between sugar intake and clinical caries outcomes (Sheiham and James, 2014; Bernabe et al., 2016; Moores et al., 2022), and others have conceptually described the ecological plaque hypothesis (Lamont et al., 2018), none has yet integrated three critical domains into a single actionable framework: (i) comparative multi-species metabolic responses to different sugar types; (ii) the ecological transition from antagonistic to synergistic microbial networks under sugar stress, including cross-kingdom interactions; and (iii) a stratified prevention model that explicitly maps public health interventions to the specific ecological disruptions they target.

In this review, we fill this gap by proposing an integrated ecological-functional framework that links mechanistic microbiology with translational prevention. We demonstrate that sugar is not merely a passive substrate for acid production but a master ecological driver that modifies gene expression, EPS synthesis, intracellular storage, and cross-kingdom synergies. The objective of this study is to establish a theoretical framework and practical guidelines for the precise prevention of dental caries, moving beyond pathogen−targeted approaches towards function−targeted and personalized strategies supported by very recent evidence (Dame-Teixeira et al., 2026; Radhamanalan, 2026).

To operationalize this integrated ecological−functional framework, we have structured the review around the following logic: first, we examine sugar−specific biochemical pathways; second, we analyze how different sugars alter the behavior of key cariogenic microorganisms; thirdly, we explore the ecological consequences of these changes, including cross−kingdom interactions and suppression of commensals; and finally, we map these mechanistic and ecological insights to specific public health prevention strategies. A synthesis of this framework is presented in **Table 1**, which explicitly links each sugar exposure pattern, microbial functional change, ecological consequence, disease stage, and corresponding preventive strategy.

**Table 1:** Comparative effects of different dietary sugars on microbial metabolism and cariogenicity.

Sugar type/exposure pattern	Key microbial functional change	Ecological consequence	Disease stage	Corresponding preventive strategy(s)
Sucrose (high frequency/high concentration)	*S. mutans*: ↑EPS synthesis *(*glucans, fructans*)*, ↑IPS storage, ↑*gtfB/C/D*, ↑*gbpB/C, ↑*acid production via glycolysis (Shemesh et al., 2007; Zhang et al., 2022; Sztajer et al., 2014)	EPS matrix formation → enhanced adhesion & biofilm cohesion; pH drop <5.5 → selection of acidogenic/aciduric species (Bernabe et al., 2016; Du et al., 2022)	Initiation & early progression (white spot lesions)	Source control: WHO sugar guidelines, sugar taxation; Chemical: Fluoride; Behavioral: Sugar substitutes (sorbitol, erythritol) (WHO, 2002; Huang et al., 2023; Hajishafiee et al., 2023; Peckham and Awofeso, 2014; O'Mullane et al., 2016)
Glucose (frequent exposure)	CCR-mediated suppression of alternative sugar metabolism; upregulation of adhesive proteins (*spaP*, *srtA*) independently of EPS; rapid lactic acid production (Shemesh et al., 2007; Bruckner and Titgemeyer, 2002)	Acidification without robust EPS matrix; favors *S. mutans* over commensals (e.g., *S. sanguinis* via H_2_O_2_ reduction) (Kreth et al., 2008)	Early progression	Source control: Reduce free glucose (e.g., SSBs, processed foods); Chemical: Fluoride; Ecological: Arginine (alkali generation) (Tenuta et al., 2023; Liu and Burne, 2009; Del Rey et al., 2025)
Fructose (moderate cariogenicity)	LevQRST system activation; *fruA* upregulation (fructan hydrolysis); less potent than sucrose in EPS formation (Zeng and Burne, 2016)	Moderate acidification; can cross-feed *C. albicans*; less dysbiotic than sucrose (Du et al., 2022; Scheinin et al., 1976)	Intermediate stage (enamel caries)	Source control: Limit high-fructose corn syrup; Ecological: Probiotics (e.g., *L. reuteri*, *S. dentisani*) may help restore balance (Hedayati-Hajikand et al., 2015; Rodriguez et al., 2016; Rippe and Angelopoulos, 2015)
Lactose (low cariogenicity)	Phospho-β-galactosidase hydrolysis; low *gtf* expression; possible IPS accumulation (Zeng and Burne, 2021; Jurakova et al., 2023)	Minimal pH drop; preserves commensal flora (e.g., *S. salivarius*); no significant dysbiosis (Woodward and Rugg-Gunn, 2020)	No to minimal disease (if part of balanced diet)	No specific intervention needed except good oral hygiene; dairy components (Ca^2+^, casein) provide protective effects (Moynihan, 2016)
Sucrose + repeated exposure (chronic)	Sustained F-ATPase upregulation (acid tolerance response); membrane fatty acid changes; enhanced cross-kingdom synergy with *C. albicans* (Baker et al., 2017; Minah et al., 1981; Johansson et al., 2016; Ellepola et al., 2019)	Persistent low pH; *Lactobacillus* and *Actinomyces* enrichment in deep lesions; alkali-generating commensals suppressed (Gross et al., 2010; Wen et al., 2022; Campus et al., 2025; Kreth et al., 2008)	Established dentin caries & root caries	Ecological regulation: Arginine-containing formulations (ADS activation); Targeted: Probiotics/prebiotics; Chemical: High-concentration fluoride varnish (Yin et al., 2026; Del Rey et al., 2025; Zhang et al., 2020; O'Mullane et al., 2016)
Any sugar (extreme frequency)	Collapse of buffering capacity; remineralization cannot keep pace; Stephan curve fails to recover (Liu et al., 2022; Cho et al., 2023)	Net mineral loss → cavitation; irreversible biofilm dysbiosis with polymicrobial synergy (bacteria + *C. albicans*)	Cavitated lesion/advanced caries	Restorative treatment (filling/crown) plus secondary prevention: strict sugar control, fluoride, ecological modulation (arginine, xylitol)
Sugar-free/sugar-limited	Dominance of alkali-generating species (*S. sanguinis*, *S. gordonii*); ADS activity maintains pH > 6.0 (Zhu et al., 2018; Liu and Burne, 2009)	Symbiotic biofilm; remineralization exceeds demineralization	Health/caries-free state	Maintenance: Low free-sugar diet, fluoride toothpaste, regular hygiene; Optional: Prebiotics (arginine) for high-risk individuals (Yu et al., 2021; Caglar et al., 2005)

This table summarizes the major metabolic characteristics of commonly consumed dietary sugars and their effects on biofilm formation, ecological balance, and cariogenic potential. Differences in extracellular polysaccharide synthesis, acid production, and microbial ecological shifts associated with each sugar type are highlighted.

This is a narrative (non-systematic) review, aimed at synthesizing current knowledge on sugar−driven cariogenic mechanisms, microbial ecology, and public health strategies. It does not follow a formal systematic review protocol.

## 2 Biochemical basis of carbohydrate metabolism and caries initiation

Sucrose, as a disaccharide composed of fructose and glucose, is a major constituent of table sugar and is commonly found in processed foods like white sugar (Giacaman, 2018). Although sucrose consists of two monosaccharides, comparisons of plaque composition reveal that sucrose is significantly more cariogenic than a combination of glucose and fructose (Cury et al., 2000). Both the frequency of exposure and the concentration of sucrose consumed strongly influence its cariogenicity, with frequency generally regarded as the more critical determinant (Moynihan and Kelly, 2014). The incidence of dental caries is positively correlated with sucrose concentration within a specific range (Newbrun, 1982). Increasing both the concentration and frequency of sugar intake can lower dental plaque matrix pH, clinically resulting in a higher incidence of dental caries (Bernabe et al., 2016; Pitts et al., 2021).

Glucose is the most common monosaccharide in nature, serving as the primary carbon source for microbial survival and a component of blood sugar (Vieira Lima et al., 2023). Epidemiological studies examining the relationship between glucose and caries are limited. However, evidence indicates that diabetic patients are more susceptible to root surface caries due to elevated blood glucose levels in saliva and gingival fluid (Vieira Lima et al., 2023; Moore et al., 2001). Moreover, adolescents with diabetes are at approximately twice the risk of dental caries compared to their non-diabetic counterparts (Beheshti et al., 2021). Fructose, an isomer of glucose, is primarily found in fruits and honey (Rippe and Angelopoulos, 2015). It is significantly less cariogenic than sucrose, as demonstrated by the classic Turku sugar studies, which reported a notable reduction in DMFT scores (Scheinin et al., 1976). Lactose is a β-1,4-linked disaccharide composed of galactose and glucose, primarily found in dairy products like milk (Ugidos-Rodriguez et al., 2018). Lactose has a relatively low acid production capacity and is considered the least cariogenic among common dietary sugars. However, it is classified as moderately cariogenic (Zeng and Burne, 2021; Woodward and Rugg-Gunn, 2020). Compared to sucrose, the minor changes in caries biofilm flora induced by lactose are believed to contribute to the relatively small reduction in plaque pH associated with lactose (Shi et al., 2020). Milk contains about 5% lactose, but milk intake is negatively correlated with the increase in caries incidence, mainly due to the caries-preventive effects of calcium-phosphorus molecules and casein in milk (Woodward and Rugg-Gunn, 2020).

### 2.1 Metabolic pathways of fermentable carbohydrates in the oral cavity

#### 2.1.1 Glycolysis and lactic acid production: core biochemical reactions for caries

Glycolysis is a critical metabolic pathway in the oral cavity, particularly with regard to fermentable carbohydrates such as sucrose and glucose. When these sugars are metabolized by bacteria that cause tooth decay, such as *Streptococcus mutans*, they undergo glycolysis, leading to the production of lactic acid. Sucrose, glucose, fructose, and lactose enter bacterial cells through the phosphotransferase system (PTS) (Ajdic and Pham, 2007). Glucose is internalized by enzyme II (EII) on the cell membrane of *S. mutans*, and is phosphorylated to form glucose-6-phosphate (Glu-6P), which then enters the Embden-Meyerhof-Parnas (EMP) pathway (Kawada-Matsuo et al., 2016). During transport, these sugars are phosphorylated to glucose-6-phosphate (G6P) by phosphoenolpyruvate (PEP) before entering either the EMP or the Pentose Phosphate Pathway (PPP) (Willenborg and Goethe, 2016). When fructose is transported via PTS, it generates fructose-1-phosphate (F-1-P) and fructose-6-phosphate (F-6-P), which are subsequently converted to fructose-1,6-bisphosphate (F-1,6-bP) by phosphofructokinase and enter glycolysis (Zeng et al., 2018). Lactose is hydrolyzed by phosphorylated-β-galactosidase to release glucose, which is re-internalized by the PTS and eventually generates pyruvate and NADPH (Zeng and Burne, 2021). Pyruvate produced from glycolysis is converted into lactate by lactate dehydrogenase (Willenborg and Goethe, 2016). Sucrose can also be transported by the MSM system, an ABC transporter system, but it requires activation by melibiose and raffinose (Vadeboncoeur and Pelletier, 1997). Sucrose transported via the MSM system enters the cytoplasm in a non-phosphorylated form. This stands in stark contrast to the PTS pathway, in which sucrose is phosphorylated to sucrose-6-phosphate during transport; non-phosphorylated sucrose is hydrolyzed intracellularly by sucrase into free glucose and free fructose (Zeng and Burne, 2016).

Carbon Catabolite Repression (CCR) significantly regulates carbon metabolism and virulence in *S. mutans* by preferentially controlling carbohydrate utilization. Glucose acts as the optimal carbon source for carbon control protein A (CcpA), which inhibits sugar metabolism proteins and reduces the efficiency of the PTS system for transporting other sugars when glucose is present (Zeng et al., 2023; Bruckner and Titgemeyer, 2002). As a monosaccharide, glucose is more easily absorbed by microorganisms than disaccharides such as sucrose. When sucrose and glucose are present together, glucose inhibits the expression of genes related to sucrose-dependent adhesion and biofilm formation. This is evidenced by a reduction in biofilm thickness when glucose is added in the presence of sucrose (Shemesh et al., 2007). Nevertheless, both sucrose and glucose can promote *S. mutans* adhesion, primarily due to their ability to upregulate surface adhesion protein genes (*spaP* and *srtA)*, which mediate sucrose-independent adhesion (Shemesh et al., 2007; Esberg et al., 2017; Jiang et al., 2006). Although glucose and fructose are isomers, the altered transcriptome of *S. mutans* grown in fructose compared to glucose suggests that these two sugars are not metabolized equally, with fructose metabolism inhibited in the presence of glucose (Zeng et al., 2018).

In addition to lactic acid, a variety of other organic acids and metabolic by-products produced by dental plaque microorganisms contribute to plaque acidification and caries development. During carbohydrate fermentation, oral bacteria metabolize sugars through glycolysis and ancillary pathways, generating not only lactate but also formate, acetate, pyruvate, and other short-chain organic acids. These acids lower the local pH within the biofilm, particularly when substrate availability is high and saliva buffering is limited, thereby promoting demineralization of enamel and facilitating caries progression (Yu et al., 2026). Such metabolic diversity reflects the complex biochemical output of mixed microbial communities in plaque, where multiple acidogenic taxa contribute to a sustained acidogenic environment.

Although lactic acid is often the most abundant fermentation end product due to the activity of strongly acidogenic species like *Streptococcus mutans*, evidence from plaque metabolic profiling indicates that other organic acids significantly contribute to the acid load and demineralization within dental biofilms (Ganas and Schwendicke, 2019). The accumulation of these various acidic metabolites synergistically depresses plaque pH below the critical threshold (~5.5) for enamel demineralization, highlighting the multifactorial nature of microbial acid production in cariogenesis.

#### 2.1.2 Extracellular polysaccharide synthesis: formation of sucrose and glucans/fructans

Another crucial aspect of carbohydrate metabolism in the oral cavity is the synthesis of extracellular polysaccharides. Sucrose is used by *S. mutans* and other cariogenic bacteria to produce glucans and fructans. These polysaccharides contribute to the structural integrity of dental biofilms, enhancing bacterial adhesion to tooth surfaces and helping bacteria resist removal by salivary flow (Lin et al., 2021; Jakubovics et al., 2021). The formation of EPS not only fosters a cariogenic environment, but also promotes acid retention produced during fermentation, further exacerbating enamel demineralization. Therefore, understanding the mechanisms of EPS synthesis and its implications for caries development is essential for developing effective preventive strategies.

### 2.2 Acid tolerance responses and microenvironmental adaptation in cariogenic biofilms

Beyond carbohydrate metabolism and EPS synthesis, the capacity of cariogenic microorganisms to withstand and remain metabolically active under acidic conditions is a critical determinant of caries progression. This capability is mediated by acid tolerance responses (ATR), a coordinated set of physiological and molecular adaptations that enable bacteria to maintain intracellular pH homeostasis and sustain glycolytic activity in low-pH environments (Senneby et al., 2017; Welin-Neilands and Svensater, 2007).

A central component of ATR is the F-ATPase proton extrusion system, which actively expels protons from the cytoplasm to counteract acidification. In Streptococcus mutans, upregulation of F-ATPase activity under acidic conditions enhances aciduricity and supports continued metabolic activity within cariogenic biofilms (Baker et al., 2017). In addition, membrane adaptation plays a complementary role, as alterations in fatty acid composition decrease proton permeability, thereby stabilizing intracellular pH.

At the regulatory level, global stress response networks and two-component systems (TCS) orchestrate gene expression changes that enhance acid resistance, modulate metabolic flux, and promote long-term survival under environmental stress (Baker et al., 2017). These coordinated responses enable cariogenic bacteria not only to survive but also to remain functionally active in acidic niches.

In contrast, certain commensal microorganisms contribute to alkali generation, which partially counterbalances acidification and supports ecological stability. The arginine deiminase system (ADS), widely present in oral streptococci, metabolizes arginine to produce ammonia, thereby elevating local pH and mitigating acid stress (Liu and Burne, 2009). This highlights the dynamic interplay between acidogenic and alkali-generating pathways in shaping biofilm ecology.

Importantly, these processes occur within structurally complex biofilms characterized by pronounced microenvironmental heterogeneity, including spatial gradients of pH, oxygen, and ionic composition. Such gradients create localized ecological niches that selectively favor acidogenic and aciduric populations while driving site-specific demineralization at the tooth surface (Von Ohle et al., 2010). This spatial and functional heterogeneity is a defining feature of cariogenic biofilms and underscores the importance of considering both microbial physiology and biofilm architecture in understanding disease progression.

### 2.3 Acid production and pH dynamics: the clinical significance of the Stephan curve

The Stephan curve illustrates the relationship between sugar consumption, acid production and subsequent changes in oral pH. Following the intake of fermentable carbohydrates, the oral pH can fall below the critical threshold of 5.5, leading to enamel demineralization. This dynamic is clinically significant because it underscores the importance of dietary habits and oral hygiene in preventing tooth decay. The curve also depicts the recovery phase, during which salivary buffering and remineralization processes can restore pH levels. However, frequent acid challenges from sugar consumption can hinder this recovery process, increasing the risk of caries. Therefore, understanding these pH dynamics is vital for developing effective dietary recommendations and preventive measures against dental caries (Liu et al., 2022).

### 2.4 Spatial heterogeneity of dental biofilms and its role in cariogenesis

Dental plaque biofilms are not uniform structures; instead, they exhibit significant spatial heterogeneity that is critical for understanding caries pathogenesis. Sugar metabolism within biofilms generates localized acidic niches as acidogenic bacteria such as *Streptococcus mutans* and *Lactobacillus* ferment sugars, pro While this review centers on microbial metabolic responses during organic acids like lactic acid (Xiao et al., 2017). These acidic zones are typically confined to regions near bacterial colonies, where pH gradients form within the biofilm. The pH in these localized areas can drop significantly, creating microenvironments with low pH that favor the growth of acid-tolerant species while inhibiting the growth of more pH-sensitive organisms.

Such microbial selection within the biofilm is essential for cariogenesis, as the proliferation of acidogenic and aciduric bacteria accelerates enamel demineralization. In addition to pH gradients, nutrient gradients also emerge due to differential sugar metabolism, where regions with higher bacterial density consume available nutrients more quickly, thereby promoting the formation of an acidic environment (Hwang et al., 2016). This spatial variability within the biofilm allows for the development of highly localized regions of low pH that facilitate the initiation and progression of carious lesions.

### 2.5 Disruption of the demineralization and remineralization dynamic balance

**Figure 1:**
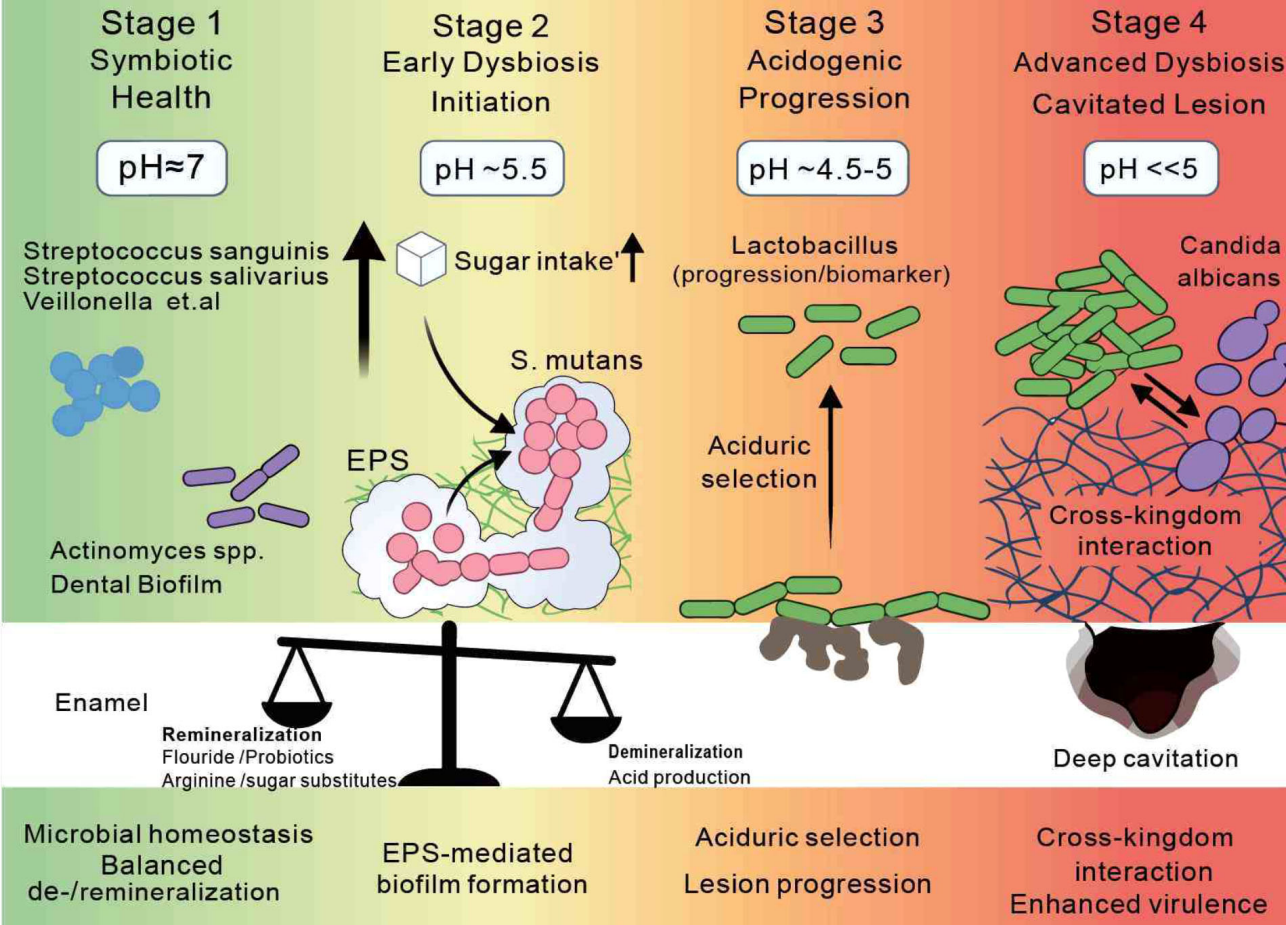
Ecological model of sugar-driven dental caries progression. Dental caries is a biofilm-mediated and ecology-driven disease characterized by dynamic shifts in the oral microbiota in response to frequent sugar intake. In the symbiotic state, the microbial community is dominated by non-*mutans streptococci* and *Actinomyces* spp., maintaining a balanced demineralization-remineralization equilibrium. Frequent exposure to fermentable carbohydrates drives acid production and extracellular polysaccharide (EPS) formation, promoting the emergence of *Streptococcus mutans* as a key driver of early dysbiosis. Sustained acidification selects for acidogenic and aciduric microorganisms, including *Lactobacillus* spp., which are associated with lesion progression and serve as biomarkers of advanced caries. In later stages, cross-kingdom interactions, particularly involving *Candida albicans*, further enhance biofilm virulence and structural stability. This model highlights that dental caries results from ecological imbalance within a polymicrobial community rather than the activity of a single pathogen.

The dynamic balance between demineralization and remineralization is essential for maintaining dental health. Factors such as frequent sugar intake, poor oral hygiene, and inadequate salivary flow can create an acidic environment that favors the growth of acidogenic bacteria while inhibiting non-cariogenic species. Consequently, the balance between demineralization and remineralization is disrupted, promoting the progression of dental caries. When the pH drops due to acid production from bacterial metabolism, hydroxyapatite crystals in enamel dissolve, resulting in mineral loss. Conversely, remineralization occurs when calcium and phosphate ions are redeposited into the enamel, facilitated by saliva and fluoride. Disruptions to this balance, particularly in environments with high sugar exposure and low pH levels, can result in irreversible damage and caries progression (**Figure 1**). This highlights the importance of integrated preventive strategies that promote remineralization while controlling acid production (Yeung et al., 2023).

## 3 The direct impact of sugars on key cariogenic microorganisms

Before presenting the evidence, it is important to note the hierarchical nature of the studies cited. Most detailed mechanistic insights-including sugar transport, gene regulation, EPS synthesis, and acid tolerance responses-are derived from *in vitro* biofilm models or animal (rodent) experiments. Observational human studies provide associative evidence linking specific microorganisms to caries prevalence or progression. Where available, controlled clinical trials or longitudinal cohort studies provide stronger causal or predictive evidence. To clarify these distinctions, we use explicit qualifiers such as: “*in vitro* evidence suggests,” “clinical studies have demonstrated,” and “epidemiological associations indicate”.

### 3.1 *Streptococcus mutans*: a key driver in early cariogenic biofilm formation

Although *Streptococcus mutans* has long been regarded as a primary cariogenic pathogen, accumulating evidence indicates that it functions within a “complex microbial community”, and its role should be interpreted in the context of ecological interactions rather than as a sole etiological agent (Chen et al., 2020). *S. mutans* is a primary contributor to dental caries due to its ability to utilize sucrose for synthesizing extracellular glucans and producing metabolic acids that lower pH (Lemos et al., 2019). These acidic conditions create favorable environments for other pathogenic microorganisms to thrive. Additionally, the sustained acid production and exceptional acid resistance of *S. mutans* establish it as the dominant strain (Lemos et al., 2019). Studies have shown differences in bacterial growth among various sugars, with sucrose inducing a higher quantity of biofilm bacteria compared to glucose and fructose (Rozen et al., 2004). The growth of planktonic bacteria was more pronounced with sucrose than with lactose or glucose, and no significant difference was found between lactose and glucose (Jurakova et al., 2023). This bacterium efficiently metabolizes sugars, particularly sucrose, through a well-adapted mechanism that leads to high acid production. This acid production lowers the pH in the oral cavity and promotes demineralization of tooth enamel. The ability of *S. mutans* to form biofilms is significantly enhanced by its production of EPS, which facilitates adherence to dental surfaces and contributes to plaque maturation. The synthesis of EPS is primarily driven by glucosyltransferases, which utilize sucrose to create adhesive glucans, thereby effectively increasing the bacterial load and enhancing the cariogenic potential of the biofilm (Liu et al., 2023, Liu et al., 2022).

#### 3.1.1 Sugar uptake and efficient acid production mechanism

The mechanisms responsible for sugar uptake in *S. mutans* are critical for its cariogenicity. *In vitro* studies have shown that this bacterium employs a phosphotransferase system (PTS) for sugar uptake, which facilitates rapid fermentation of carbohydrates into lactic acid, significantly lowering the pH in the oral cavity. The metabolic pathways activated during sugar fermentation drive the demineralization of tooth enamel, thereby establishing a direct mechanistic link between sugar intake and caries progression. Quantitative *in vitro* measurements demonstrate that *S. mutans* can produce up to 1.5 moles of lactic acid per mole of glucose, demonstrating its potent acidogenic potential (Liu et al., 2023). Mechanistically, this acid production contributes to local pH reduction and enamel demineralization in experimental models. However, clinical extrapolation of these precise yields requires caution, as the oral environment includes buffering saliva and competing microbial species.

#### 3.1.2 Sucrose utilization for extracellular glucans synthesis and biofilm maturation mechanism

The conversion of sucrose into extracellular glucans is a hallmark of *S. mutans*’ virulence. The capability of *S. mutans* to synthesize EPS from sucrose is critical for its adherence to tooth surfaces and biofilm maturation. This matrix enhances the stability of the biofilm and provides a reservoir for nutrients, further promoting the survival of *S. mutans* in acidic environments. The production of glucans not only facilitates bacterial adhesion but also reinforces the structural integrity of the biofilm, enhancing its resistance to antimicrobial agents and host defenses (Jiang et al., 2025). Consequently, the consumption of sucrose directly influences the cariogenic potential of *S. mutans* through biofilm formation and maturation (Jiang et al., 2025; Zhan et al., 2023).

The two-component signal transduction systems (TCS) are important signaling pathways that regulate the virulence of *S. mutans* and its response to environmental stimuli (Zu et al., 2019). VicRK and GcrR play crucial roles in regulating polysaccharide metabolism in *S. mutans*, while ComDE primarily modulates virulence and acid tolerance (Zu et al., 2019; Zhang et al., 2022). Specifically, the VicRK system positively regulates the synthesis of EPS, which enhances the cariogenic potential of biofilms formed by *S. mutans* (Zhang et al., 2022). Upon sucrose exposure, the expression of the *vicR* gene is upregulated, alongside increased activity of the *gcrR* promoter, leading to altered expression of associated target genes (Zhang et al., 2022). Their downstream genes mainly regulate the metabolism of IPS and EPS. The enzyme glucosyltransferase (Gtf) plays a pivotal role in this process by converting sucrose into glucans, which form a protective matrix around the bacterial community. *S. mutans* synthesizes glucans to enhance its adhesion to tooth surfaces, forming a robust biofilm that serves as a protective habitat for the bacteria. The enzymes involved in this process are glucosyltransferase B (GtfB), glucosyltransferase C (GtfC), glucosyltransferase D (GtfD) and fructosyltransferase (Ftf) (Hoshino and Fujiwara, 2022).

The role of sugars in regulating the virulence genes of *S. mutans* is controversial. Nevertheless, evidence suggests that adding sucrose or glucose promotes the expression of factors associated with tooth decay, such as *gtfB*, *gtfC*, *gtfD* and *ftf* (Shemesh et al., 2007). In contrast, lactose or excessive glucose inhibits the synthesis and activity of Gtfs, leading to reduced water-soluble glucan (WSG) production. Additionally, the increase in FTF expression induced by sucrose is lower than that induced by glucose and fructose (Rozen et al., 2004; Jurakova et al., 2023; Decker et al., 2014; Hamilton et al., 1979). The glucan-binding protein (GBP) genes *gbpB* and *gbpC*, which play a role in regulating biofilm formation, cell wall integrity and virulence, are upregulated by sucrose or glucose (Jurakova et al., 2023; Shemesh et al., 2007). This upregulation contributes to enhanced bacterial virulence. In contrast, lactose downregulates the expression of these genes.

#### 3.1.3 Sucrose utilization for intracellular glucans synthesis

In the oral cavity, some sugars are converted into EPS, while others are internalized and converted into IPS by the enzyme glycogen synthase (Glg) (Kawada-Matsuo et al., 2016). Glg serves as an energy reserve for the bacterium and functions alongside the production of lactate and pyruvate (Kawada-Matsuo et al., 2016; Busuioc et al., 2009). IPS are glycogen polymers linked by 1,6-α- and 1,4-α-glycosidic bonds, providing an endogenous source of carbohydrates for microorganisms during periods of nutrient restriction (Costa Oliveira et al., 2021). When energy is deficient, IPS are broken down by Glg enzymes to release glucose-6-phosphate (G-6-P) (Costa Oliveira et al., 2021). IPS can contribute to caries formation by prolonging the exposure of tooth surfaces to organic acids and maintaining a low pH in the plaque matrix (Busuioc et al., 2009). *S. mutans* cultured in sucrose exhibited continuous increases in *glg* manipulator expression and IPS production, whereas *glg* gene expression decreased in the presence of glucose and lactose (Jurakova et al., 2023; Costa Oliveira et al., 2021). Sucrose-induced *S. mutans* biofilms exhibit increased porosity, which facilitates acid diffusion (Cury et al., 1997). Concurrently, the content of inorganic ions such as calcium, phosphorus and fluoride decreases, which is negatively correlated with sucrose concentration (Cury et al., 1997). The adherence of *S. mutans* to hydroxyapatite discs increases with rising sucrose concentrations (0.45% to 2.4%), but declines beyond this threshold; the reasons for this are unclear (Cai et al., 2016). No sufficient evidence was found regarding the role of fructose and lactose in the adhesive capacity of *S. mutans*.

#### 3.1.4 Metabolism of other sugars in *S. mutans*

Fructanase A (FruA) hydrolyses fructans into fructose, which in turn prolongs acid production and enhances bacterial virulence (Zeng and Burne, 2008). The primary fructose-responsive regulatory system in *S. mutans* is the four-component LevQRST system, which can be activated by free fructose or fructose produced intracellularly from sucrose metabolism (Zeng and Burne, 2016). The expression of *fruA* is influenced by types of carbohydrates: glucose and fructose significantly upregulate its expression, whereas sucrose and lactose have the opposite effect (Jurakova et al., 2023). *S. mutans* exhibits growth memory behavior, as demonstrated by distinct lag phases when transferred from glucose or fructose-containing media to lactose-containing media (Zeng and Burne, 2021). It has been demonstrated that phosphorylated hexose intermediates generated during glucose or fructose metabolism act as effector molecules that bidirectionally regulate lactose utilization in *S. mutans* by modulating the DNA-binding activity of the LacR repressor.

Collectively, the above evidence reveals that different sugars exert distinct and sometimes opposing effects on gene expression, EPS/IPS accumulation, and acid production in *S. mutans*. This sugar−specific regulatory hierarchy, governed by systems such as CCR and LevQRST, provides a key mechanistic insight that distinguishes our review from prior studies, which often treated all fermentable carbohydrates as functionally equivalent. We further extend this comparative analysis in **Table 1**, which systematically contrasts sucrose, glucose, fructose, and lactose across nine functional parameters: acidogenicity, EPS yield, IPS storage, *gtf* upregulation, GBP expression, fructanase activity, biofilm porosity, adhesion threshold, and cross feeding potential.

### 3.2 Lactobacilli: the acid-resistant ‘acid producers’

The relationship between the genus Lactobacilli and dental caries is complex. Cross−sectional and longitudinal observational studies have increasingly associated Lactobacilli with caries progression rather than primary initiators (Zhang et al., 2018). *In vitro* experiments demonstrate that these bacteria exhibit strong acidogenic and aciduric properties, allowing them to thrive in the low pH environments established during the early stages of caries development (Wen et al., 2022). These bacteria thrive in acidic environments and are capable of producing lactic acid from fermentable carbohydrates, contributing to enamel demineralization. Certain species, such as *Lactobacillus salivarius* and *Lactobacillus acidophilus*, contribute to the development of caries due to their ability to produce acids and resist acidic conditions, synthesize extracellular polysaccharides and facilitate the adhesion of cariogenic bacteria (Piwat et al., 2012; Arasu et al., 2016). *Lactobacillus plantarum* and *Lactobacillus rhamnosus* are widely used for the prevention of dental caries. They inhibit *Streptococcus mutans* and other bacteria, are acid-resistant, and can colonize the oral cavity, demonstrating their potential as probiotics to promote oral health (Zhang et al., 2020; Rodriguez et al., 2016).

#### 3.2.1 The association of lactobacilli with caries progression, especially root caries

Importantly, Lactobacilli are often regarded as “biomarkers of advanced lesions,” as their abundance correlates with lesion depth and severity rather than disease initiation (Gross et al., 2010). Consequently, their role is primarily linked to the progression and maintenance of cariogenic conditions. Moreover, Lactobacilli serve as indicators of caries progression, contributing to accelerated tooth demineralization during the later stages of the disease (Kolenbrander et al., 2010; Takahashi and Nyvad, 2011; Aas et al., 2008). Higher counts of Lactobacilli in saliva and dental plaque correlate with a greater incidence of caries, emphasizing their role as indicators of caries risk, particularly in elderly populations susceptible to root caries (Soundaram et al., 2024, Roeder et al., 2026). Consuming sucrose increases the proportion of Lactobacilli in dental plaque and significantly lowers the pH of dental biofilms (Minah et al., 1981). This pH reduction disrupts the microbial balance and promotes the growth of cariogenic species (Minah et al., 1981). When cultured with sucrose, glucose and fructose at equal concentrations as the sole carbon sources, Lactobacilli exhibited superior growth on glucose and fructose compared to sucrose (Almstahl et al., 2013). After 24 hours, the pH of the glucose cultures was lower than that of the sucrose cultures, possibly because glucose is more readily available in its monosaccharide form.

#### 3.2.2 Strong acid production and acid resistance characteristics in deep carious lesions

Lactobacilli exhibit potent acidogenic activity, which is particularly detrimental in deep carious lesions. These bacteria not only contribute to the acidification of the oral environment but also enhance the cariogenic potential of biofilms through their metabolic activities. Lactobacilli can produce acids even in the presence of fluoride, which complicates caries management strategies. Their acid resistance allows them to thrive in low pH environments, further perpetuating the cycle of demineralization and caries progression. This resilience not only promotes their survival but also increases their competitive advantage over other microorganisms, including beneficial species, thereby contributing to the dysbiosis often observed in carious lesions (Liu et al., 2023). The metabolic pathways involved in acid production by Lactobacilli are crucial for understanding their role in caries development and potential therapeutic interventions (Yu et al., 2021; Roeder et al., 2026).

Clinical observational studies have shown that *Lactobacillus* abundance correlates more strongly with root caries progression and deep dentin lesions than with initial coronal enamel caries (Gondo et al., 2024; Wen et al., 2022). In ECC, *Lactobacillus* becomes prominent only in cavitated lesions, serving as a biomarker of disease severity rather than initiation (Gross et al., 2012). These phenotype−specific associations should be considered when designing preventive interventions. Recent longitudinal studies have further established *Lactobacillus* abundance as a predictor of lesion progression rather than initiation, supporting their role as ecological biomarkers of advanced dysbiosis (Dame-Teixeira et al., 2026).

### 3.3 *Actinomyces*: from symbiotic commensal to cariogenic agent

*Actinomyces* species, traditionally viewed as commensals in the oral cavity, can transition to pathogenic roles under certain conditions, particularly in the presence of high-sugar diets. *Actinomyces* species function primarily as early colonizers of the tooth surface and play a significant role in root caries initiation, particularly in aging populations (Johansson et al., 2016; Krithikadatta and Krishnan, 2024). It has been found that sucrose acts as a potentiator rather than a dependent factor in the adhesion of *Actinobacillus viscosus* and *Actinobacillus neuii* (Zhu, 1992). These bacteria can still adhere without sucrose, but its presence enhances their adhesion, and it is hypothesized that this may be due to the production of EPS by *Actinomyces* (Zhu, 1992). Their role in the oral microbiome is complex, as they can both support oral health by maintaining microbial diversity and contribute to disease during dysbiosis (Campus et al., 2025; Liu et al., 2023).

The consumption of fermentable carbohydrates significantly influences the dynamics of the microbial community in the oral cavity, particularly affecting *Actinomyces* species. High sugar intake can promote the growth of *Actinomyces* specie, leading to a shift from a healthy periodontal microbiome to a cariogenic one. This transition is characterized by increased acid production and biofilm formation, both of which contribute to the onset and progression of dental caries. Understanding these dynamics is crucial for developing effective strategies for caries prevention (Campus et al., 2025; Liu et al., 2023). This species adhere to tooth surfaces and form biofilms that facilitate the colonization of other cariogenic bacteria, such as *S. mutans*. Its capacity to metabolize sugars and produce acids contributes to the demineralization of tooth structure, rendering it a significant contributor to the carious process, particularly on root surfaces, which are more susceptible to decay (Campus et al., 2025; Malin et al., 2024).

### 3.4 Phenotype−specific microbial ecology: early childhood caries versus coronal caries versus root caries

Dental caries is not a single disease but encompasses distinct clinical phenotypes that differ in microbial composition, ecological drivers, and age-related host factors. Early childhood caries (ECC) predominantly affects primary teeth in young children. Both metagenomic and culture-based studies have consistently shown that ECC-associated biofilms are enriched in *Streptococcus mutans*, *Candida albicans*, *S. vestibularis*, and *S. salivarius*, with frequent cross-kingdom interactions between *S. mutans* and *C. albicans* that enhance EPS matrix formation and acidogenicity (Gross et al., 2012; Chalmers et al., 2015; Du et al., 2022). The thin, less mineralized primary enamel and the child’s inability to perform adequate oral hygiene independently make ECC particularly sugar−sensitive (Ugolini et al., 2023).

Coronal caries in permanent teeth (in adolescents and adults) shows a more gradual ecological transition. While *S. mutans* remains a key initiator, the biofilm is more diverse, with *Lactobacillus* species becoming prominent only in advanced dentin lesions. Commensal streptococci *S. sanguinis* and *S. gordonii*, can persist for extended periods compared with ECC-associated strains, provided that sugar exposure remains moderate (Diaz-Garrido et al., 2020).

Root caries occurs predominantly in older adults with gingival recession or periodontitis, exposing root cementum which is less mineralized and more susceptible to acid demineralization than enamel. Microbiomic studies have identified *Actinomyces* species (*A. naeslundii, A. viscosus*) and *Lactobacillus* as dominant taxa in root surface caries, with a comparatively lesser role for *S. mutans* than in coronal caries (Gondo et al., 2024; Johansson et al., 2016). The root surface biofilm is further shaped by variations in salivary flow-often reduced in older adults-and by proximity to gingival crevicular fluid, which can partially buffer pH while simultaneously providing nutrients for proteolytic bacteria.

Preventive strategies targeting *S. mutans* such as xylitol, fluoride are highly relevant for ECC and coronal caries, whereas interventions that modulate *Actinomyces* and *Lactobacillus* for example arginine−based alkali generation and improved biofilm disruption, may be more critical for root caries prevention. This phenotype−specific understanding is now reflected in **Table 1** by distinguishing disease stages.

## 4 Sugar-mediated oral microbial ecological interactions

### 4.1 *Candida albicans* involvement: fungal-bacterial cross-kingdom alliance

Sugar exposure does not merely enrich acidogenic species; it actively remodels the architecture of microbial interaction networks. In healthy biofilms, commensals such as *S. sanguinis* and *S. gordonii* produce hydrogen peroxide and alkali, creating an antagonistic environment that limits cariogen overgrowth (Bowen et al., 2018). However, sustained sucrose availability shifts this balance towards synergistic cross−kingdom alliances, most notably between *S. mutans* and *Candida albicans.*

*Candida albicans* is the most common opportunistic pathogenic fungus and is frequently detected in early childhood caries (ECC) (Sztajer et al., 2014). Cross−sectional and longitudinal studies have shown that *C. albicans* is detected in up to 90% of severe ECC cases but in less than 25% of caries−free preschool children, indicating a strong phenotype−specific association (Sztajer et al., 2014). This synergy with *S. mutans* is less pronounced in adult coronal caries and rarely reported in root caries, suggesting that cross−kingdom interactions are particularly relevant to the rapid progression seen in ECC. *C. albicans* has limited caries-causing potential on its own. However, *Candida albicans* acts primarily as a “facilitator of cariogenic virulence” through cross-kingdom interactions with bacteria such as *Streptococcus mutans*. Rather than functioning as an independent cariogenic pathogen, it enhances biofilm structural integrity and acidogenicity, particularly in ECC (Sztajer et al., 2014). Studies have shown that *C. albicans* enhances the cariogenic potential of *S. mutans* by promoting biofilm formation and increasing acid production when exposed to fermentable sugars (Kim et al., 2020b). This synergistic effect leads to a more virulent biofilm structure that is resistant to conventional treatments (Liu et al., 2023).

#### 4.1.1 Synergistic cariogenic effects of *Candida albicans* and *Streptococcus mutans*

The GtfB enzyme of *S. mutans* plays a key role in this interaction: when GtfB binds to *C. albicans*, glucan is produced, promoting further *S. mutans* colonisation (Sztajer et al., 2014). *C. albicans* can utilize glucose as a carbon source, but it is inefficient at metabolizing sucrose. In contrast, *S. mutans* can break down sucrose into glucose and fructose, providing *C. albicans* with energy and promoting its growth (Du et al., 2022). Sucrose alters the adhesion pattern between *S. mutans* and *C. albicans*, promoting co-adhesion. When sucrose is limited, *C. albicans* tends to co-adhere with *S. gordonii*, whereas co-adhesion with *S. mutans* increases sixfold when dextran is produced (Du et al., 2022; Ellepola et al., 2019). *C. albicans* not only contributes to the acidogenic environment through its metabolic activities but also facilitates the adherence of *S. mutans* to dental surfaces via the production of extracellular polysaccharides (Kim et al., 2020b).

Recent multi−omics analyses have demonstrated that, in mixed−species biofilms, sucrose−fed *S. mutans* provides glucose and fructose to *C. albicans*, while *C. albicans* enhances the stability of the EPS matrix. This cross-kingdom interaction establishes a positive feedback loop, increasing biofilm virulence by more than threefold compared with mono-species biofilms (Du et al., 2022; Ellepola et al., 2019). This synergistic transition is a hallmark of sugar−driven ecological dysbiosis and represents a conceptual advance over earlier models that considered each species separately.

#### 4.1.2 Impact of sugary environments on *Candida* colonization and biofilm formation

High sugar intake promotes the growth of *C. albicans* by providing the necessary substrates for its metabolism, leading to increased biofilm density and virulence. This biofilm not only protects the fungal cells from host immune responses but also enhances the cariogenic potential of the entire microbial community. Consequently, managing sugar consumption is pivotal in controlling *C. albicans* colonization and mitigating its impact on oral health (Roeder et al., 2026).

Collectively, these findings underscore that different microorganisms assume distinct ecological roles throughout the stages of caries development, functioning as initiators, facilitators, or biomarkers within a dynamic microbial network, rather than as isolated pathogens.

### 4.2 Expanding the cariogenic microbiome: beyond *Streptococcus mutans*

Recent advances in metagenomics and microbial ecology have expanded the list of taxa implicated in dental caries beyond the classical focus on *Streptococcus mutans*. *Streptococcus sobrinus*, a closely related mutans streptococcus, frequently co-exists with *S. mutans* in caries associated communities and is correlated with increased caries risk (Wakamatsu et al., 2026). *S. sobrinus* demonstrates potent acidogenicity and acid tolerance, contributing to enhanced plaque acidification and disease progression, particularly when present in conjunction with other cariogenic bacteria (Wakamatsu et al., 2026).

In addition to traditional acid producing streptococci, *Selenomonas sputigena* (*S. sputigena*), a Gram negative anaerobe previously associated with periodontal disease, has been identified as a key partner in cariogenic biofilms (Hawkes et al., 2026). Multi-omics analyses and experimental models have shown that *S. sputigena* becomes incorporated into plaque biofilms with *S. mutans*, forming structured multicellular assemblies. These assemblies enhance acid production and biofilm virulence, exacerbating enamel demineralization and caries severity *in vivo*, even though *S. sputigena* alone is insufficient to induce caries (Cho et al., 2023).

## 5 Non-cariogenic *Streptococci*: ecological defense mechanisms and their suppression

Non-cariogenic streptococci, such as *Streptococcus salivarius*, are crucial in maintaining oral health by competing with cariogenic pathogens and producing antimicrobial substances such as H_2_O_2_. *S. sanguinis* is a non-cariogenic oral commensal that usually establishes itself during the initial stages of biofilm formation. *S. gordonii* is also one of the earliest colonizers of the oral cavity. These beneficial bacteria contribute to the ecological balance of the oral microbiome by inhibiting the growth of pathogenic species like *S. mutans*. However, elevated sugar levels can suppress these protective bacteria, thereby increasing the risk of caries (Tenuta et al., 2023). The transition from a healthy oral microbiome to a cariogenic one is characterized by significant changes in microbial networks and interactions. In a balanced state, beneficial bacteria outnumber pathogens, thereby maintaining oral health. However, factors such as high sugar intake can disrupt this balance, leading to an increase in cariogenic species such as *S. mutans* and *C. albicans*. This ecological imbalance facilitates the progression of dental caries and complicates treatment strategies. Understanding these microbial networks is critical for developing interventions aimed at restoring a healthy oral microbiome (Dame-Teixeira et al., 2026).

### 5.1 Ecological imbalance: transition from healthy to cariogenic microbiota

High-sugar diets exert significant pressure on the ecological niches occupied by non-cariogenic *streptococci*, which often leads to their decline and the proliferation of cariogenic species. *S. gordonii* is one of the earliest colonizers of the oral cavity. It has an extracellular dextran structure similar to that of *S. mutans* and its dextran synthase is primarily regulated by *gtfG* (Rgg). The addition of sucrose upregulates *gtfG* expression in *S. gordonii* (Lozano et al., 2019). *In vitro* studies demonstrate that sucrose promotes the aggregation of *S. gordonii* (Tanzer et al., 2008). *S. gordonii* uses sucrose to synthesize extracellular glucan for adhesion. Although *S. gordonii* can colonize teeth in the absence of sucrose, sucrose enhances its colonization rather than being essential for it (Tanzer et al., 2008). The primary caries protective factors of *Streptococcus sanguinis* include glucosyltransferases (GTFs), encoded by the *gtfP* gene, and catalase, encoded by *spxB* (pyruvate oxidase). The primary caries protective factors of *Streptococcus sanguinis* include glucosyltransferases (GTFs), encoded by the *gtfP* gene, and catalase, encoded by *spxB* (pyruvate oxidase). In *S. sanguinis*, GTFs promote the synthesis of glucans that facilitate stable commensal biofilm formation on tooth surfaces, thereby occupying ecological niches and limiting colonization by highly cariogenic species. In parallel, catalase generates hydrogen peroxide, which exerts antimicrobial effects that inhibit the growth and virulence of cariogenic bacteria such as *S. mutans* (Zhu et al., 2018). In the presence of sucrose, *gtfP* expression was significantly increased, while no significant change in *spxB* expression was observed (Lozano et al., 2019; Diaz-Garrido et al., 2020). Co-culturing *S. mutans* with *S. sanguinis* and *S. gordonii* in the presence of glucose or sucrose reduces the proportion of *S. sanguinis*, slows its growth rate, and decreases hydrogen peroxide production (Kreth et al., 2008). Consequently, shifts in microbial populations driven by sugar consumption can create an environment that is conducive to dental caries (Malin et al., 2024). The degree to which sugar suppresses commensal alkali−generating streptococci may vary by caries phenotype. In ECC, young children often have higher sugar consumption frequency and less mature biofilms, leading to rapid outgrowth of *S. mutans* and near−elimination of *S. sanguinis*. In root caries, older adults may have reduced salivary flow and exposed root surfaces, where the protective function of *S. sanguinis* is compromised not only by sugar but also by age−related changes in the oral environment (Giacaman, 2018; Dame-Teixeira et al., 2026). Therefore, phenotype−specific ecological dynamics should inform targeted probiotic or prebiotic strategies.

Recent high throughput sequencing and multi omics analyses have provided robust evidence that the transition from a healthy to a cariogenic microbiota reflects broad community level changes, rather than the activity of a single pathogen. Comparative microbiome studies reveal distinct compositional profiles between health and disease, with caries associated plaques typically showing increased relative abundance of acidogenic/aciduric taxa such as *Neisseria, Lautropia*, *Lactobacillus*, *Porphyromonas*, and *Aggregatibacter*, accompanied by reduced representation of health associated commensals (Li et al., 2023). Metagenomic and beta-diversity analyses consistently demonstrate significant separation between healthy and caries-active communities, indicating an overall ecological shift (i.e., dysbiosis) during the progression of dental caries (Dong et al., 2025).

Beyond bacteria, evidence for oral mycobiome alterations has begun to emerge. Fungal members such as *Candida* spp. are detectable in supragingival biofilms and show differential abundance patterns in subjects with caries, suggesting that fungi may contribute to community interactions, acidification dynamics, or biofilm architecture that favor cariogenesis (Xiang and Liu, 2026). Although mycobiome research in caries is still nascent, recent reviews highlight the potential role of fungal taxa in modulating oral biofilm ecology and caries risk, especially in childhood populations.

Functionally, caries associated communities also exhibit enrichment in metabolic pathways related to carbohydrate utilization, acid production, and stress responses, further supporting the notion that the healthy oral microbiome transitions toward a cariogenic state through both compositional and functional dysbiosis (Li et al., 2023).

### 5.2 Alkaline production and competitive inhibition by symbiotic bacteria

The production of alkali by non-cariogenic streptococci is a key mechanism by which they protect against dental caries. These bacteria metabolize amino acids and other substrates to produce ammonia, which increases oral pH, counteracting the acidification caused by cariogenic bacteria. Additionally, they inhibit the growth of *S. mutans* through competitive exclusion, effectively limiting the resources available for cariogenic pathogens. When bacterial growth rates were measured with different carbon sources, *S. mutans* grew less in glucose than in sucrose, while the opposite pattern was observed for *S. sanguinis* (Mansouri et al., 2023). The competitive inhibition exerted by these beneficial bacteria is crucial in preventing the establishment of cariogenic biofilms. However, the high sugar environment can diminish their competitive edge, allowing cariogenic bacteria to thrive (Liu et al., 2022). This dual action of alkali production and competitive inhibition highlights the vital role of symbiotic *streptococci* in maintaining oral health and preventing caries (Liu et al., 2023).

Importantly, recent work has identified “microbiological dysbiosis scars”-persistent shifts in commensal gene expression even after sugar removal-which may explain caries recurrence (Dame-Teixeira et al., 2026). The major microorganisms involved in sugar-driven ecological dysbiosis and dental caries are summarized in **Table 2**, including both cariogenic species and health-associated commensal bacteria.

**Table 2:** Major cariogenic and health-associated microorganisms discussed in this review.

Microorganism	Major characteristics	Role in caries development	Ecological significance
*Streptococcus mutans*	Strong acidogenicity, acid tolerance, extracellular polysaccharide (EPS) synthesis	Core cariogenic pathogen responsible for acid production, biofilm maturation, and enamel demineralization	Dominates under sugar-rich and low-pH conditions, driving ecological dysbiosis
*Streptococcus sobrinus*	Potent acidogenicity and aciduricity	Enhances plaque acidification and increases caries risk, particularly in co-existence with *S. mutans*	Functions synergistically within cariogenic microbial communities
*Lactobacillus* spp.	Highly acidogenic and aciduric	Associated with lesion progression and maintenance of acidic environments	Thrives in advanced low-pH cariogenic niches
*Candida albicans*	Opportunistic fungal species capable of interacting with bacterial biofilms	Enhances EPS matrix formation and promotes *S. mutans* adherence and virulence	Represents a cross-kingdom synergistic partner in cariogenic biofilms
*Selenomonas sputigena*	Gram-negative anaerobe incorporated into structured biofilms	Enhances biofilm virulence and enamel demineralization in mixed-species communities	Acts as a pathobiont contributing to polymicrobial cariogenic assemblies
*Streptococcus gordonii*	Early colonizing commensal streptococcus	Competes with cariogenic species under low-sucrose conditions	Associated with oral microbial homeostasis
*Streptococcus sanguinis*	Health-associated early colonizer	Competes with *S. mutans* and is associated with reduced cariogenicity	Supports ecological stability and oral health
*Streptococcus salivarius*	Alkali-generating commensal species	Contributes to pH buffering and inhibition of cariogenic microorganisms	Health-associated bacterium with probiotic potential
*Streptococcus vestibularis*	Low-virulence oral commensal species	Limited direct involvement in caries development	Associated with maintenance of microbial stability and oral health

This table summarizes the principal microorganisms involved in sugar-driven ecological changes within dental biofilms, including their major biological characteristics, roles in caries development, and ecological significance. Both classical cariogenic species and newly recognized polymicrobial contributors are included, together with representative health-associated commensal microorganisms involved in maintaining oral microbial homeostasis.

## 6 Public health strategies for caries prevention based on microbial ecology theory: source control and chemical intervention

### 6.1 World health organization sugar intake guidelines

Reducing the frequency and amount of free sugar intake is a cornerstone of caries prevention. Strong epidemiological and clinical evidence demonstrates a dose-response relationship between sugar consumption and caries risk (Sheiham and James, 2014). The World Health Organization (WHO) has established guidelines recommending that free sugars should comprise less than 10% of total energy intake. Further health benefits are observed at lower levels, ideally below 5% (Moores et al., 2022). The aim of these guidelines is to combat the high prevalence of dental caries and other non-communicable diseases associated with excessive sugar consumption. The WHO emphasizes the importance of reducing sugar intake, particularly in children, to prevent dental caries and promote overall health. The guidelines are supported by extensive research linking high sugar intake to dental caries, obesity and other health issues. Implementing these guidelines requires coordinated efforts from governments, health organizations and communities to create environments that facilitate healthier dietary choices.

### 6.2 Remineralization and antimicrobial ecological effects of fluoride

#### 6.2.1 Regulation of demineralization/remineralization balance by fluoride

Among preventive strategies, fluoride use and dietary sugar control remain the most effective and evidence-based approaches for caries prevention. Fluoride enhances remineralization, inhibits demineralization, and suppresses bacterial metabolism, with extensive clinical evidence supporting its efficacy across populations (Peckham and Awofeso, 2014). Fluoride plays a critical role in maintaining the balance between demineralization and remineralization. It enhances the remineralization process by promoting the formation of fluorapatite, which is more resistant to acid attack than hydroxyapatite (Yu et al., 2017). Fluoride’s ability to inhibit the activity of cariogenic bacteria, such as *Streptococcus mutans*, also contributes to its protective effects against caries development. The incorporation of fluoride into dental products such as toothpaste and varnishes, is widely recognized as an effective way of preventing caries and facilitating enamel repair. Ongoing research continues to explore the most effective concentrations and delivery methods of fluoride to maximize its benefits while minimizing the risks associated with excessive exposure.

#### 6.2.2 Inhibition of microbial metabolism and acid production by fluoride ions

Mechanistic *in vitro* studies have shown that fluoride ions inhibit the metabolism of cariogenic bacteria, including disruption of enolase and F−ATPase in *S. mutans*, thereby reducing acid production (Banerjee et al., 2024). Clinical evidence from numerous randomized controlled trials (RCTs) and systematic reviews confirms that community water fluoridation and topical fluoride applications significantly reduce caries incidence in populations (Peckham and Awofeso, 2014). Thus, while the antimicrobial mechanism is well characterized *in vitro*, the clinical efficacy of fluoride is supported by high−level causal evidence (Peckham and Awofeso, 2014). Fluoride acts as a dual ecological modulator: it reduces the frequency of pH drops by suppressing bacterial glycolysis and simultaneously promotes enamel repair, thereby increasing resilience against sugar challenges (O'Mullane et al., 2016).

### 6.3 Multi-level public health strategies for caries prevention: integrating government, community, and individual interventions

Restricting sugar intake is currently one of the most evidence-based public health strategies for preventing dental caries worldwide. Achieving this goal requires coordinated action at the government, community, and individual levels; a single measure alone is unlikely to yield lasting results. At the government level, sugar taxes on sugary beverages have effectively reduced consumption in Mexico, the United Kingdom, and Chile, though direct evidence linking taxation to a reduction in dental caries remains limited (Hajishafiee et al., 2023). Supporting policies should include mass media campaigns, front-of-package labeling, and the establishment of standards for sugar content in foods. At the community level, community water fluoridation and workplace health programs collectively create a supportive environment, thereby reducing the behavioral burden on individuals. At the individual level, efforts should focus on raising health awareness regarding hidden sugars, actively reducing sugar intake, using sugar substitutes, and maintaining good oral hygiene.

## 7 Public health strategies for caries prevention based on microbial ecology theory: ecological regulation and emerging methods

### 7.1 Probiotics and prebiotics: reshaping healthy oral microbiota

The application of probiotics and prebiotics in oral health have gained attention as a strategy to modulate the oral microbiome. *In vitro* and animal studies have demonstrated that specific strains such as *Lactobacillus* and *Bifidobacterium*, can inhibit *S. mutans* through competitive exclusion and production of bacteriocins (Caglar et al., 2005). RCTs have shown that certain probiotic formulations reduce salivary *S. mutans* and *Lactobacillus* spp. counts (Cortes-Dorantes et al., 2015; Villavicencio et al., 2018; Inchingolo et al., 2025). However, the clinical evidence is currently moderate, with heterogeneity across strains, doses, and delivery methods. Long−term effectiveness and translation into population−level caries reduction remain to be firmly established. Unlike fluoride which reduces overall metabolic activity, probiotics and prebiotics aim to restore ecological balance without eliminating specific species. This functional, rather than taxonomic, targeting is a key distinction of the ecological approach (Luo et al., 2024).

#### 7.1.1 Application of specific lactobacilli and *Lactobacillus reuteri* strains

The use of specific Lactobacilli strains, particularly *Lactobacillus reuteri*, has been highlighted for their potential in caries management. These probiotics can produce antimicrobial substances that inhibit pathogenic bacteria, enhance the immune response, and modulate the oral microbiome’s composition. Studies have demonstrated that *L. reuteri* can effectively reduce levels of *S. mutans* and improve overall oral health. Furthermore, the application of these strains in various forms, such as lozenges or dairy products, has shown to be feasible and effective in clinical settings, suggesting a practical approach to caries prevention through probiotic supplementation (Hedayati-Hajikand et al., 2015; Rodriguez et al., 2016). Emerging strategies move beyond live probiotics. Postbiotics such as heat−killed *Lactobacillus* or their metabolic products and bacteriophages targeting *S. mutans* offer precise ecological modulation without long−term colonization (Radhamanalan, 2026).

#### 7.1.2 The promoting effect of arginine and other prebiotics on alkaline bacteria

Arginine, a naturally occurring amino acid, has been identified as a potent prebiotic that promotes the growth of alkalinogenic bacteria in the oral cavity. This growth is crucial as these bacteria can neutralize acids produced by cariogenic pathogens, thereby reducing the risk of enamel demineralization. Research indicates that arginine supplementation can lead to increased salivary pH and enhanced remineralization of early carious lesions. Additionally, other prebiotics, such as inulin and fructooligosaccharides, have shown similar effects in promoting beneficial microbial populations, highlighting the importance of dietary components in maintaining oral health and preventing caries (Yu et al., 2021). Very recent clinical trials have confirmed that arginine−containing dentifrices significantly increase salivary pH and reduce caries increment in high−risk children, with the effect mediated by the arginine deiminase system (Del Rey et al., 2025; Yin et al., 2026).

### 7.2 Targeted strategies against cariogenic microorganisms

The development of targeted strategies against cariogenic microorganisms represents a significant advancement in caries prevention. These strategies include the use of non-cariogenic sweeteners, antimicrobial peptides, and enzyme inhibitors that specifically disrupt the pathogenicity of cariogenic bacteria while preserving beneficial oral flora. For example, the use of non-cariogenic sweeteners, such as xylitol, has been demonstrated to reduce *S. mutans* levels, whereas antimicrobial peptides can directly inhibit bacterial growth and biofilm formation. Moreover, research on enzyme inhibitors targeting glucosyltransferases-key enzymes in biofilm synthesis-offers promising avenues for the development of innovative caries management strategies (Atta et al., 2025).

#### 7.2.1 Alternative therapies: non-cariogenic sweeteners

Mechanistic *in vitro* studies demonstrate that xylitol is not fermented by cariogenic bacteria and, at high concentrations, inhibits *S. mutans* growth via a futile PEP−PTS cycle and downregulation of gtf genes (Jurakova et al., 2023). Randomized controlled trials have shown that regular use of xylitol−containing products can reduce *S. mutans* levels, but clinical evidence for a direct reduction in caries increment is limited and inconsistent (Cocco et al., 2017; Soderling and Pienihakkinen, 2025; Ortiz-Saez et al., 2024). Therefore, while the mechanistic basis for sugar substitutes is sound, their clinical caries−preventive efficacy should not be equated with that of fluoride without stronger evidence.

#### 7.2.2 Advances in antimicrobial peptides, enzyme inhibitors, and vaccine development

Antimicrobial peptides, enzyme inhibitors such as Gtf inhibitors, and anticaries vaccines are active areas of preclinical and early−phase research. *In vitro* and animal model studies have demonstrated that these agents can selectively target cariogenic bacteria or disrupt biofilm formation. Furthermore, host genetic variation, including differences in FUT2 and AMY1 copy number, affects salivary oligosaccharide composition and may influence individual susceptibility to sugar-driven dysbiosis (Kamitaki et al., 2026). Incorporating such biomarkers into personalized caries risk assessment is a frontier area. These approaches are currently in experimental or early translational stages and require further validation before clinical implementation. Taken together, while ecological and microbiome-based interventions offer promising avenues for caries prevention, they should be viewed as complementary strategies that enhance, rather than replace, established measures such as fluoride use and dietary sugar control. The major prevention strategies discussed in this review, together with their mechanisms, evidence levels, clinical applicability, and limitations, are summarized in **Table 3**.

**Table 3:** Caries prevention strategies: mechanisms, evidence level, clinical applicability, and limitations.

Prevention strategy	Mechanism	Evidence level	Clinical applicability	Limitations
Fluoride	Remineralization, antibacterial	Strong	Widely implemented	Overexposure risk (Peckham and Awofeso, 2014)
Sugar substitutes	Metabolic inhibition	Moderate	Adjunctive	Dose-dependent (Liang et al., 2024)
Arginine	Alkali generation	Moderate	Adjunctive	Individual differences (Del Rey et al., 2025)
Probiotics	Microbiome modulation	Moderate	Adjunctive	Limited long-term data (Inchingolo et al., 2025)
Vaccines	Immune targeting	low	Clinical evidence insufficient	Safety concerns (Kumar et al., 2025)

This table summarizes representative prevention strategies for dental caries discussed in this review, including their primary mechanisms of action, current level of supporting evidence, clinical applicability, and major limitations. The strategies are presented within the context of the ecological-functional framework proposed in this review, highlighting approaches that target microbial metabolism, ecological dysbiosis, and biofilm-associated cariogenic processes.

## 8 Knowledge gaps and future directions

### 8.1 Remaining questions in phenotype−specific microbial ecology

Although we have integrated phenotype-specific features, including ECC, coronal caries, and root caries, into the main mechanistic sections, several important questions remain unanswered. First, it remains unclear whether the functional thresholds for sugar-induced dysbiosis differ between primary and permanent enamel or between enamel and root cementum. Second, longitudinal studies tracking the same individuals from ECC to permanent dentition are lacking, leaving it unknown whether an “ECC-prone” microbiome persists after the transition to mixed dentition. Third, the optimal design of phenotype-specific interventions, such as arginine formulations for root caries versus anti-Candida strategies for ECC, requires direct comparative clinical trials. Addressing these gaps will be essential for advancing precision caries prevention.

### 8.2 Role of food matrix and sugar exposure patterns

During dietary intake, sugar exists not in isolation but in a complex form interacting with other nutrients; this complex system of coexistence is referred to as the “food matrix” (Weaver and Givens, 2025; Mulet-Cabero et al., 2024). While the role of free sugars in caries development is well established, the impact of food matrix and patterns of sugar exposure remains incompletely understood. Research indicates that the texture and form of ingested food can influence energy metabolism and the duration of food retention in the mouth, and therefore affect the duration of microbial substrate exposure within the oral cavity (Forde and Bolhuis, 2022). Sugar-sweetened beverages have been demonstrated to possess cariogenic potential, attributed not only to their high sugar content but also to their intrinsic acidity (Valenzuela et al., 2021). In contrast, naturally occurring sugars in cereals, vegetables, and milk do not contribute significantly to caries development, largely due to the protective effects of dietary fiber, calcium ions, and other buffering components within the food matrix (Moynihan, 2016).

### 8.3 Multi-omics, spatial microbiology, and function-targeted prevention

Recent advances in multi-omics technologies, including metagenomics, metatranscriptomics, and metabolomics, have provided new insights into the functional dynamics of oral microbial communities. These approaches enable the identification of active metabolic pathways and microbial interactions beyond taxonomic composition (Nascimento et al., 2017; Dong et al., 2025). In parallel, research in spatial microbiology has shown that biofilms are highly complex ecosystems in which pH levels, bacterial arrangement, and metabolites vary across different stages of dental caries; these factors play a critical role in the development of dental caries (Kim et al., 2020a). Future research integrating multi-omics with spatial analysis is expected to provide a more comprehensive understanding of cariogenic biofilms.

Traditional methods of caries prevention primarily target specific pathogens, particularly *Streptococcus mutans*. However, since caries is not caused by a single bacterial species, it is more effective to focus on preventing caries by addressing functional characteristics such as biofilm formation, acid production, and acid resistance than by simply suppressing the presence of a single bacterial species (Zhu et al., 2023). This has led to a paradigm shift toward function-targeted interventions, aiming to modulate microbial activities and restore ecological balance rather than eliminate specific organisms. Such strategies may offer more sustainable and broadly effective approaches to caries prevention.

### 8.4 Microbiome- and metabolite-based diagnostic potential for sugar-driven caries

Beyond therapeutic and preventive strategies, growing evidence suggests that sugar-induced changes in the oral microbiome and metabolome may serve as potential diagnostic markers for caries risk assessment and early detection. Frequent sugar exposure drives reproducible ecological shifts toward acidogenic and aciduric microbial communities, characterized by increased abundance of taxa such as Streptococcus, *Lactobacillus*, and other low-pH-adapted organisms. These compositional changes are accompanied by alterations in metabolic output, including elevated levels of formate, lactate, proline, and glycine, which collectively contribute to sustained plaque acidification (Kim et al., 2023).

Recent advances in metagenomic and metabolomic profiling have demonstrated that such microbial and metabolic signatures for caries progression such as *Streptococcus mutans*, *Veillonella atypica* and severe caries *such as Prevotella denticola and Campylobacter SGB19347* can distinguish between caries-active and caries-free individuals, and may even detect early dysbiotic transitions prior to the onset of clinically visible lesions. In particular, functional analyses have revealed enrichment of pathways related to carbohydrate metabolism, acid production, and stress tolerance in caries-associated biofilms, suggesting that functional biomarkers may be as informative as taxonomic shifts (Zhang et al., 2025).

Saliva- and plaque-based analyses therefore represent promising non-invasive approaches for caries risk prediction, enabling earlier and more personalized preventive interventions (Liu et al., 2025). Although these approaches are still evolving, they highlight the translational potential of understanding sugar-driven microbial ecology in the development of precision diagnostics for dental caries.

### 8.5 Personalized caries risk assessment and precision prevention

Comprehensive risk management and personalized prevention strategies are essential for effective caries control. By integrating individual risk factors, such as dietary habits, oral hygiene practices, and genetic predispositions, healthcare providers can tailor prevention strategies to meet the specific needs of patients. This personalized approach may include recommendations for dietary modifications, targeted use of probiotics and prebiotics, and regular dental check-ups. Moreover, the incorporation of advanced diagnostic tools, such as salivary biomarkers, can enhance risk assessment and enable timely interventions, ultimately improving oral health outcomes and reducing the incidence of caries (Sonmez et al., 2025).

### 8.6 From conceptual framework to clinical translation

While our integrated ecological−functional framework provides a novel synthesis, its clinical utility requires validation through prospective studies that assess both sugar exposure patterns and multi-kingdom biofilm characteristics. The application of AI-driven spatial microbial mapping on tooth surfaces represents a promising approach to correlate *in situ* microbial ecology with individual caries risk (Li et al., 2025).

While this review centers on microbial metabolic responses to dietary sugars, it is important to recognize that the clinical manifestation of caries is shaped by a constellation of host and environmental factors. Salivary factors, including flow rate and buffering capacity, modulate plaque pH and microbial community stability, thereby influencing the persistence of acidogenic conditions (Nyvad and Takahashi, 2020). Fluoride exposure affects both enamel resistance and bacterial metabolism (Ten Cate, 2013). Anatomical features such as pits and fissures provide sheltered niches for biofilms, and oral hygiene practices determine plaque accumulation (Pang et al., 2021). Moreover, host immune responses can regulate microbial colonization and inflammatory sequelae (Marsh and Zaura, 2017). Although these determinants are beyond the scope of this review, they interact with microbiome ecology in complex ways and are well summarized in specialized reviews.

## 9 Conclusion

The mechanisms underlying sugar-induced dental caries involve multiple interconnected processes within the complex microbial ecosystem of the oral cavity. This review reveals that sugar is not merely a substrate for acidogenic bacteria, but also a pivotal environmental factor that modulates the metabolic activity, adhesion properties, microbial interactions and overall community structure of key microorganisms, such as *Streptococcus mutans*, Lactobacilli species, *Actinomyces* and *Candida albicans*. This modulation drives the transition of the oral microbiome from a healthy state to one that is conducive to caries development.

Given the complexity of dental caries, its prevention must evolve beyond traditional antibacterial or acid-neutralizing strategies. An ecological regulation approach focusing on maintaining the balance of the oral microbiome is essential. A robust public health framework for caries prevention must be multi-layered and integrative. It should address sugar consumption at a policy level, promote the widespread use of fluoride in communities and explore ecological modification techniques, such as probiotics and prebiotics, at an individual level.

Several limitations of this review should be acknowledged. First, and most importantly, many of the mechanistic insights discussed-including sugar transport, gene regulation, EPS synthesis, and acid tolerance responses are derived primarily from *in vitro* biofilm models or animal studies. While these provide valuable mechanistic understanding, their direct extrapolation to human caries development or prevention requires caution. Second, the observational human studies cited in this review provide associative, rather than causal, evidence. Randomized controlled trials are available for fluoride, certain probiotics, and xylitol. We have explicitly indicated the corresponding strength of evidence for these interventions. Third, there is significant heterogeneity among studies regarding sugar type, concentration, exposure frequency, biofilm models, and outcome measures, which limits direct quantitative comparisons. Fourth, emerging interventions such as postbiotics, phages, and antimicrobial peptides are supported only by preclinical evidence; we have clearly labeled them as experimental.

Elucidating the specific response mechanisms of the oral microbial network under sugar stress is crucial for future research. Understanding these interactions will pave the way for the development of targeted, innovative preventive tools that can sustainably promote oral ecological health. Fostering a more nuanced understanding of the interplay between diet, microbial ecology and caries development is a necessary step toward future reductions in caries incidence. However, translating mechanistic and associative findings into effective population-level interventions requires rigorous clinical validation. Researchers must carefully consider diverse perspectives. It is important to explicitly distinguish hypothesis-generating mechanistic studies from hypothesis-testing clinical trials. Developing strategies that are both effective and sustainable depends on this careful approach. Such a holistic strategy is crucial for addressing the persistent challenges posed by dental caries in contemporary society.
